# Non-infectious Complications of Common Variable Immunodeficiency: Updated Clinical Spectrum, Sequelae, and Insights to Pathogenesis

**DOI:** 10.3389/fimmu.2020.00149

**Published:** 2020-02-07

**Authors:** Hsi-en Ho, Charlotte Cunningham-Rundles

**Affiliations:** ^1^Department of Medicine, Icahn School of Medicine at Mount Sinai, New York, NY, United States; ^2^Department of Pediatrics, Icahn School of Medicine at Mount Sinai, New York, NY, United States

**Keywords:** common variable immunodeficiency, autoimmunity, immune dysregulation, thrombocytopenia, hemolytic anemia, granulomatous disease, interstitial lung disease, enteropathy

## Abstract

Non-infectious complications in common variable immunodeficiency (CVID) have emerged as a major clinical challenge. Detailed clinical spectrum, organ-specific pathologies and associated sequelae from 623 CVID patients followed in New York since 1974 were analyzed, and recent insights to pathogenesis were reviewed. Non-infectious manifestations were present in 68.1% of patients, and they do not tend to be present in isolation. They include autoimmunity (33.2%), chronic lung disease (30.3%), lymphoid hyperplasia/splenomegaly (20.9%), liver disease (12.7%), granulomas (9.3%), gastrointestinal disease (7.3%), lymphoma (6.7%), and other malignancies (6.4%). In the lungs, interstitial disease and bronchiectasis were the most common findings, with lymphoma at this site being a rare (*n* = 6), but serious, manifestation. Bronchiectasis was not a prerequisite for the development of interstitial disease. In the liver, granulomas and nodular regenerative hyperplasia were the most common. Gastrointestinal disease may affect any segment of the intestinal tract, with lymphoid infiltrations and villous blunting being the leading histologic findings. With progression of organ-specific diseases, a wide spectrum of associated sequelae was observed. Lymphoma was more common in females (*P* = 0.036)—all B cell types except in one subject. Solid organ transplantations (liver, *n* = 5; lung, *n* = 4; combined lung and heart, *n* = 2) and hematopoietic stem cell transplantations (for B cell lymphoma, *n* = 1) have rarely been performed in this cohort, with mixed outcomes. Recent identification of monogenic defects, in ~10–30% of various CVID cohorts, has highlighted the molecular pathways that can affect both antibody production and broader immune regulation. In addition, cellular defects in both innate and adaptive immune systems are increasingly recognized in this syndrome.

## Introduction

Common variable immunodeficiency (CVID) is considered a primary defect, characterized by reduced serum levels of immune globulin (Ig) G, IgA, and or IgM, with reduced or absent specific antibody production (1–4). The diagnosis excludes secondary causes of hypogammaglobulinemia and other well-defined primary immunodeficiencies, including combined immunodeficiencies. It was first recognized by Sanford et al. in 1954 (5). With an estimated prevalence of 1:50,000–1:25,000, it is the most common symptomatic primary immunodeficiency. CVID patients share a central failure in B cell differentiation into functional Ig-secreting plasma cells, and while classified among B-cell defects, an increasing number of cellular defects have been recognized in recent years.

The clinical spectrum of CVID is broad but consist of two main phenotypes: one group with predominantly recurrent infections and a second group with additional autoimmune/inflammatory manifestations. These non-infectious complications may be evident at presentation or may appear afterward, and they include progressive lung disease, autoimmunity, gastrointestinal inflammatory disease, granulomatous disease, liver disease, lymphoid hyperplasia and infiltrative disease, and the development of cancer, especially lymphoma (6–8). This phenotypic distinction has important clinical implication because the risk of death is estimated at 11 times higher for patients with non-infectious complications compared to those without (7, 9). In addition, while the introduction of Ig replacement therapy has greatly reduced the number of infections (10), it does not appear to prevent or ameliorate most inflammatory and autoimmune conditions (6, 7). Non-infectious complications in CVID are therefore emerging as a major challenge, requiring a better understanding of underlying pathogenesis and additional therapeutics.

Recent investigations of this phenotypic syndrome have led to the discovery of a number of monogenic defects, in ~10–30% of CVID patients, providing potential insights into both pathogenesis and more direct therapies (11–13). Broad innate and adaptive immune dysregulation are also increasingly identified, especially in subjects with non-infectious complications. In the clinics, rituximab has been shown to be effective for the treatment of autoimmune cytopenia in CVID (14), while additional immune modulators and biologics are becoming more widely utilized, with benefits in some cases (15–18).

Large cohort and registry data have provided key insights into non-infectious complications of CVID (6–8, 19–24). Previously, we have found that not all such complications were equally deleterious in our cohort. Increased morbidity risk is closely associated with a number of organ-specific pathologies, such as lung impairment, liver, and gastrointestinal disease, as well as lymphoma (7). Additional cohort studies have shown that several conditions, such as autoimmunity, granulomatous infiltrations, lymphoproliferation, and enteropathy appears to be clinically interrelated, suggesting some level of shared pathogenesis (8, 19). Here, we provide an updated clinical spectrum of non-infectious complications from 623 patients with CVID followed at our center, with delineated organ-specific pathologies and associated sequelae. In addition, we highlight recent efforts in dissecting monogenic defects and dysregulated immune pathways in CVID.

## Non-Infectious Complications: Updated Clinical Spectrum and Sequelae

### Demographics and Immunologic Parameters

We detail the clinical spectrum, organ-specific pathologies, and associated sequelae from a cohort of 623 patients (277 males, 346 females) confirmed as having CVID based on standard criteria (1) in a patient cohort seen at Mount Sinai Hospital (1986–2019) and/or before this at the Memorial Sloan-Kettering Cancer Center (1974–1986). This study was approved by the Mount Sinai Hospital Institutional Review Board. The median age of symptom onset (major infection or characteristic non-infectious manifestation) was 25 years for males and 28 years for female. Consistent with prior reports, males were diagnosed with CVID earlier (median age of 30 years for males vs. 33.7 years for female). Overall, 18% of patients were diagnosed under the age of 21.

Median serum immunoglobulin levels at diagnosis were IgG, 237 mg/dL; IgA, 7 mg/dL; and IgM, 20 mg/dL. Serum IgG was <100 mg/dL in 24.7%; IgA was <7 mg/dL in 49.2%, IgM was <25 mg/dL in 55.9%. For subjects examined in this way, peripheral B cells were <1% of total lymphocytes in 7.6%; isotype switched memory B cells were <0.55% of total B cells in 35% (Table 1).

**Table 1:** Immunologic parameters.

	Normal range^*^	**Median (range)**
IgG (mg/dL)	700–1,600	237 (UD−687)
IgA (mg/dL)	70–400	7 (UD−255)
IgM (mg/dL)	40–230	20 (UD−945)
**T-cell populations**		
CD3+, % (*n* = 359)	55–89	75.5 (16–98)
CD3+, cells/mm^3^	750–2,500	1,080 (160–5,383)
CD3+CD4+, cells/mm^3^ (*n* = 254)	480–1,700	633 (76–2,828)
CD3+CD8+, cells/mm^3^ (*n* = 200)	180–1,000	381 (26–3,247)
**B-cell populations**		
CD19+, % (*n* = 410)	5–15	9 (0–58)
CD19+, cells/mm^3^	75–375	146 (0–840)
Isotype-switched memory B cells		
(CD19+CD27+IgD–), % (*n* = 223)	6.5–29.2	1 (0–29)

* *Normal range listed is for adults. UD, undetectable. Percentage of CD3+ T cells and CD19+ B cells are expressed as % of total lymphocytes; Percentage of isotype-switched memory B cells (CD19+CD27+IgD–) are expressed in percentage of total CD19+ B cell population*.

### Overview of Non-infectious Complications

In our CVID cohort, 68.1% of the patient had one or more non-infectious complications (Table 2). Consistent with prior reports (8, 20, 21), the most common manifestation was autoimmunity (33.2%, *n* = 207), with hematologic autoimmunity being the most prevalent (21.7%, *n* = 135). The most common organ-specific manifestation was functional or structural chronic lung diseases (30.3%, *n* = 189), followed by gastrointestinal diseases (17.3%, *n* = 108), and liver diseases (12.7%, *n* = 79). Lymphoid hyperplasia and/or splenomegaly was also common, with a prevalence of 20.9% (*n* = 130) in this cohort. Lymphoma was confirmed in 42 patients (6.7%), while other solid organ cancers was found in 40 patients (6.4%). Granulomatous disease was confirmed by biopsy in 58 patients (9.3%). Non-infectious complications did not tend to occur in isolation. Amongst those with such conditions, the majority (60.8%) experienced two or more non-infectious manifestations in their lifetime.

**Table 2:** Non-infectious complications.

	**No**.	**% of cohort** **(*n* = 623)**	**Chapel et al.** **(8) (*n* = 334)**	**Quinti et al.** **(20) (*n* = 224)**	**Wehr et al.** **(21) (*n* = 303)**	**Farmer et al.** **(24) (*n* = 205)**
Infection only	199	31.9	26%	NR	NR	NR
Non-infectious complication	424	68.1	74%	NR	NR	NR
Autoimmunity	207	33.2	NR	25.9%	20.3%	NR
Chronic lung disease	189	30.3	NR	46.4%	NR	NR
Lymphoid hyperplasia/splenomegaly	130	20.9	30%	26.4%^**^	40.5%^**^	25.9%^**^
Gastrointestinal disease	108	17.3	9%	22.4%^***^	NR	21.5%
Liver disease	79	12.7	9%^*^	NR	NR	9.3%
Granulomas	58	9.3	8%	NR	11.6%	20%
Lymphoma	42	6.7	3%	1.8%	NR	5%
Other malignancies	40	6.4	3%	4.5%	NR	22%^****^

* *Categorized as hepatomegaly in the original study*.

** *categorized as splenomegaly in the original studies*.

*** *categorized as chronic diarrhea in the original study*.

**** *categorized as solid organ malignancy in the original study. NR, Not reported*.

The prevalence of autoimmunity, chronic lung disease, gastrointestinal disease, and granulomatous disease was comparable to previously published cohort data (Table 2). Based on existing cohorts, the prevalence of autoimmunity ranged from 20.3 to 33.2%, chronic lung disease ranged from 30.3 to 46.4%, gastrointestinal disease ranged from 9 to 22.4%, and granulomatous disease ranged from 8 to 20%. The overall incidence of lymphoma was higher in our cohort (6.7%) compared to most prior reports (8, 20, 23, 25), though the reasons behind this are unclear.

### Chronic Lung Disease and Associated Complications

Lung failure has been a leading cause of death amongst CVID patients. The presence of functional or structural lung impairment is associated with increased mortality (hazard ratio 2.06) (7). However, not all forms of lung disease appear to be equally deleterious, as increased mortality risk has not been observed in those with radiographic evidence of bronchiectasis alone (7). It is thought that the pathogenesis of bronchiectasis may be fundamentally different than other forms of chronic lung disease in CVID, with bronchiectasis being more closely related to tissue damage from recurrent pulmonary infections rather than broader immune dysregulation (26).

**Figure 1:**
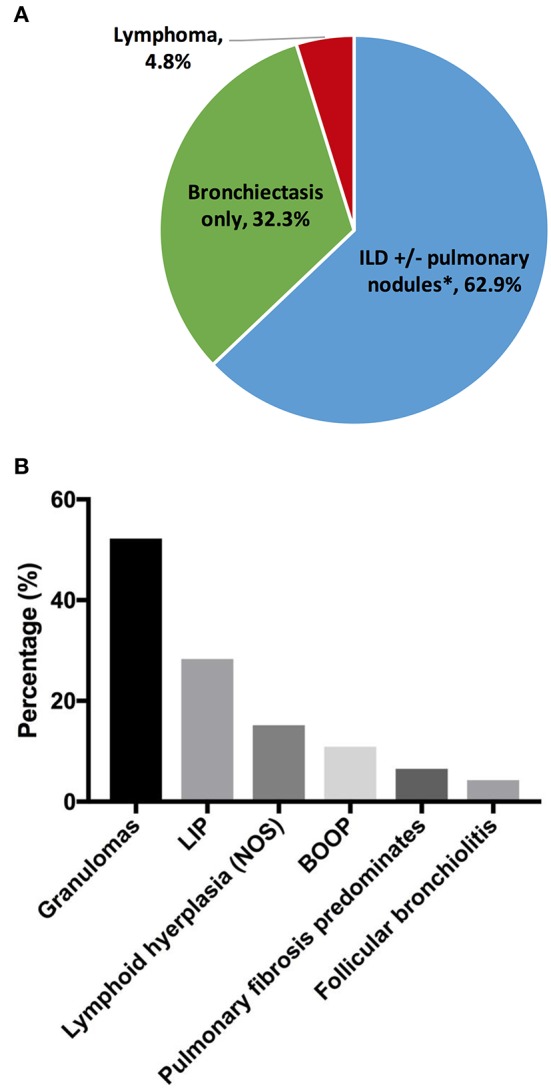
Chronic lung disease. **(A)** Lung disease types by radiographs and/or pathology reports (*n* = 124). **(B)** Interstitial lung disease pathologies (*n* = 46). *Thirteen out of 65 subjects with ILD had concurrent bronchiectasis. ILD, interstitial lung disease; LIP, lymphoid interstitial pneumonia; BOOP, bronchiolitis obliterans organizing pneumonia.

Chronic lung disease was the most common organ-specific complication in our cohort (*n* = 189, 30.3%). To provide better delineation of distinct CVID-associated lung diseases, we reviewed existing radiography and pathology reports in the cohort. Specific radiographic and/or biopsy-based diagnosis was available in 124 patients (Figure 1A). Amongst this group, the prevalence of interstitial lung disease (ILD) was 62.9% [*n* = 78; ILD was defined as computed tomography (CT) evidence of ground glass opacities with or without more than 4 pulmonary nodules or mediastinal lymphadenopathy]. Radiographic evidence of co-existing ILD and bronchiectasis was observed in 10.5% (*n* = 13) of patients with lung disease, but the majority of patients with ILD (*n* = 65) did not have concurrent CT findings of bronchiectasis, indicating that the development of ILD was independent from the presence of bronchiectasis. The prevalence of isolated bronchiectasis, based on CT findings, was observed in 32.3% (*n* = 40). Lymphoma was diagnosed by lung biopsy in 6 subjects (4.8%), highlighting the necessity of tissue diagnosis in select cases to differentiate pulmonary nodules from malignancy.

Tissue histology may be useful to guide the selection of therapeutics for the distinct forms of interstitial disease (27). Biopsy reports were available in 46 subjects with ILD (Figure 1B). Amongst the subjects in this group, the most common pathology features were lung granulomas (52.2%, *n* = 24). Some forms of lymphoid infiltration were found in 43.5% (*n* = 20) of the patients (lymphoid interstitial pneumonia, 28.3%; lymphoid hyperplasia, not otherwise specified, 15.2%). Extensive lymphoid infiltrations and granulomas may be observed concurrently in some patients (and this was specified in 6 subjects, 13%). Features of bronchiolitis obliterans organizing pneumonia were found in 10.9%, and follicular bronchiolitis was found in 4.3%. In 3 subjects (6.5%), extensive pulmonary fibrosis was the predominant finding at the time of biopsy.

Chronic lung disease may lead to significant morbidity, including progressive structural and/or functional decline, as well as chronic oxygen supplementation requirement. Further complications may also develop from either lymphocytic interstitial lung disease, granulomatous lung disease, or bronchiectasis. Pulmonary hypertension was observed in 5.3% (*n* = 10) of the subjects with lung disease. This complication may arise from diverse lung pathologies (interstitial lung disease, *n* = 2; granulomatous lung disease, *n* = 2; bronchiectasis *n* = 1; lung pathology not-specified, *n* = 5). Six of these subjects subsequently developed cor pulmonale.

Six patients underwent lung transplantation. An additional patient underwent combined lung and liver transplant, but follow-up data were unavailable. Clinical information, including primary lung disease, comorbidity, and outcome, is summarized in Table 3. Patient 1 experienced hyperacute rejection and died within 3 days of transplantation. Patient 2 experienced acute rejection and died of associated complications after 8 months. Patient 3 had known CVID-associated haplotype HLA A1-B8-DR3 and two additional family members with a CVID phenotype. She presented with low IgG, low peripheral B cells and interstitial lung disease. After lung transplantation, she experienced acute rejection and died after 1 year. Patient 4 and 5 experienced chronic rejections and died 5 and 6 years following transplantation, respectively. Patient 6 had undergone lung transplant 4 months prior to the time of report.

**Table 3:** Lung transplant outcomes.

	**Patient 1**	**Patient 2**	**Patient 3**	**Patient 4**	**Patient 5**	**Patient 6**
Age, sex	34, F	69, F	38, F	27, M	25, M	63, M
Lung pathology	Pulmonary fibrosis predominates	ILD (granuloma and lymphoid infiltrate), bronchiectasis	ILD	Chronic obstructive disease	Pulmonary fibrosis predominates	ILD
CVID-associated comorbidities	Liver disease	None	None	Enteropathy	Lymphoid hyperplasia	NRH, AIHA, ITP, lymphoid hyperplasia
Transplant procedure	Lung	Lung	Lung	Lung and heart	Lung and heart	Lung
Outcome	Died of hyperacute rejection within days	Died of acute rejection after 8 months	Died of acute rejection after 1 year	Died of chronic rejection after 5 years	Died of chronic rejection after 6 years	Alive 4 months post-transplant

*ILD, interstitial lung disease; NRH, nodular regenerative hyperplasia; AIHA, autoimmune hemolytic anemia; ITP, Immune thrombocytopenic purpura*.

### Autoimmunity

Autoimmunity was observed in 33.2% (*n* = 207) of the overall cohort (Table 4). There was no gender difference in the prevalence of autoimmunity. As reported previously, the clinical spectrum was wide and includes both hematologic (21.7%, *n* = 135) and organ-specific autoimmunity (Table 4). Immune thrombocytopenic purpura (ITP) was the most common (16.2%, *n* = 101), followed by autoimmune hemolytic anemia (AIHA, 7.7%, *n* = 48), consistent with prior cohort data (7, 8, 20). Eight patients were found to have anti-IgA antibodies. Other associated autoimmune conditions include rheumatoid arthritis (2.7%, *n* = 17) and uveitis (1%, *n* = 6). Rarer autoimmune complications (<1%) include alopecia, autoimmune thyroid disease, systemic lupus erythematosus, vasculitis, antiphospholipid syndrome, anticardiolipin antibody, psoriasis, multiple sclerosis, lichen planus, vitiligo, type 1 diabetes mellitus, and pernicious anemia. Myasthenia gravis, autoimmune pancreatitis, and severe oral aphthous ulcers (*n* = 1 each) were newly observed autoimmune conditions in CVID since our last report (7).

**Table 4:** Autoimmune manifestations.

	**No**.	**% of cohort (*n* = 623)**
Hematologic autoimmunity	135	21.7
ITP	101	16.2
AIHA	48	7.7
Evans syndrome	29	4.7
Rheumatoid arthritis^*^	17	2.7
Anti-IgA antibody	8	1.3
Uveitis	6	1.0
Alopecia	5	0.8
Autoimmune thyroid disease	5	0.8
Others^**^	<5	<0.8

* *additional 12 patients had non-specified arthritis*.

** *Others (n < 5 each): pernicious anemia, anticardiolipin antibody, antiphospholipid syndrome, type 1 diabetes mellitus, uveitis, multiple sclerosis, systemic lupus erythematosus, lichen planus, vasculitis, vitiligo, psoriasis, myasthenia gravis, autoimmune pancreatitis, severe aphthous ulcer, autoimmune pancreatitis*.

### Gastrointestinal Inflammatory Disease and Malabsorption

Gastrointestinal disease and malabsorption in CVID are associated with increased mortality (HR = 2.78 and 2.06, respectively) (7). Varying degrees of enteropathy, which can mimic inflammatory bowel disease clinically, are commonly seen, and may affect any parts of the gastrointestinal tract. In our cohort, the prevalence of gastrointestinal disease was 17.3% (*n* = 108) overall. The presentation may be severe, with malnutrition (significant nutritional deficiency and/or total parenteral nutrition requirement) recorded in 35.2% (*n* = 38) of those with gastrointestinal manifestations. Comprehensive biopsies from upper and lower endoscopies were available in 34 subjects (Table 5). Amongst this group, disease involvement in the small intestines and large intestines was seen in 79.4% (*n* = 27) and 50% (*n* = 17), respectively. Absence or near absence of plasma cells was a common feature, specified in 47.1% (*n* = 16). In the small intestine, typical histologic findings included intraepithelial lymphocytosis (64.7%, *n* = 22), villous atrophy/blunting (32.4%, *n* = 11), nodular lymphoid hyperplasia (8.8%, *n* = 3), and non-specific inflammation (8.8%, *n* = 3). Granulomas were noted in one subject. CD8+ T cell infiltrates were specified in 3 subjects and mixed cellular infiltrates (neutrophils, eosinophils, histiocytes) were specified in 3 subjects. In the large intestine, non-specific inflammation (29.4%, *n* = 10), nodular lymphoid hyperplasia (11.8%, *n* = 4), and granulomas (8.8%, *n* = 3) were most commonly reported. Crypt abscesses were seen in one subject. While some histological findings (i.e., villous blunting) may resemble celiac disease, gene expressions analysis by microarray had previously indicated that they were likely distinct disease entities (28). In our cohort, 17 subjects with gastrointestinal disease had received celiac genetics testing, and only 3 subjects were found to carry celiac-associated HLA-DQ haplotype.

**Table 5:** Gastrointestinal disease pathologies by location.

	**No**.	**% of group**
**Comprehensive biopsies available**	34	100
**Small intestine disease**	27	79.4
Intraepithelial lymphocytosis	22	64.7
Villous atrophy/blunting	11	32.4
Nodular lymphoid hyperplasia	3	8.8
Non-specific inflammation	3	8.8
Granulomas	1	2.9
**Large intestine disease**	17	50.0
Non-specific inflammation	10	29.4
Nodular lymphoid hyperplasia	4	11.8
Granulomas	3	8.8
**Gastric disease**	20	58.8
Gastritis, NOS^*^	7	20.6
Gastropathy, NOS	7	20.6
Lymphoid aggregates	3	8.8
Metaplasia	2	5.9
Granulomas	1	2.9
**Esophageal disease**	3	8.8
Eosinophilic esophagitis	2	5.9
Esophagitis, NOS	1	2.9

* *NOS, not otherwise specified*.

Gastric disease was noted in 58.8% (*n* = 20) of available reports. Gastritis and gastropathy (not otherwise specified) were seen in 7 subjects each. Lymphoid aggregates were observed in 3 subjects, and granulomas were observed in 1 subject. Some degree of metaplasia was noted in 2 subjects. From esophageal biopsies, two subjects were found to have eosinophilic esophagitis, while one patient had non-specific inflammation.

### Liver Disease and Sequelae

**Figure 2:**
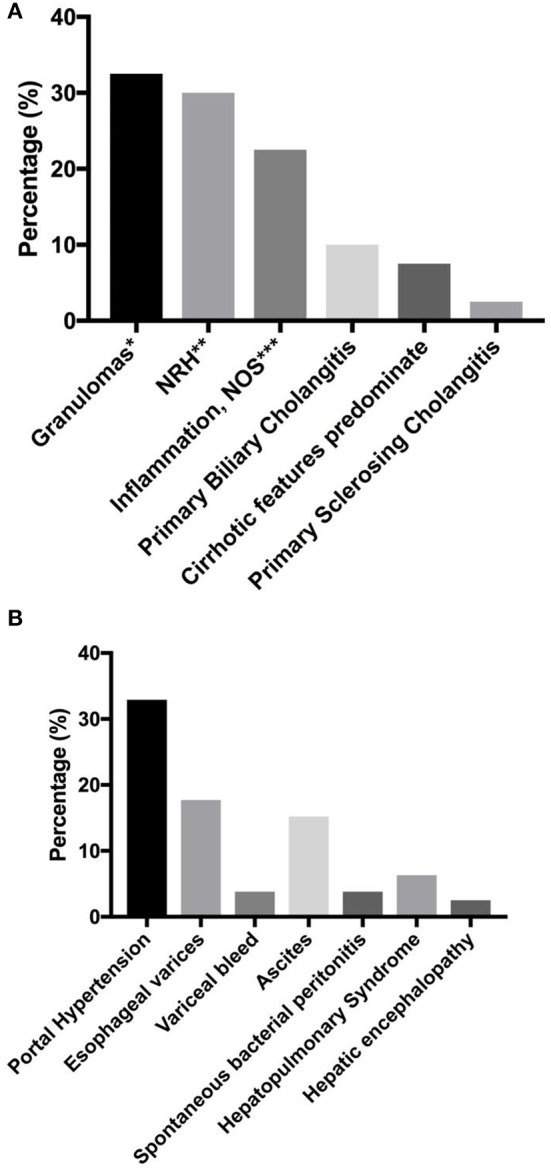
Liver disease and associated sequelae. **(A)** liver disease pathologies (*n* = 40). **(B)** Liver disease-associated sequelae, reported as percentage of total number of subjects with known liver disease (*n* = 79). *Two patients with concurrent NRH. One patient with concurrent obliterative portal venopathy specified. **One patient with concurrent obliterative portal venopathy specified. *** lymphoid infiltrates predominate in 5 patients. NRH, nodular regenerative hyperplasia; NOS, not otherwise specified.

In our previous report, we found that the presence of liver disease was also associated with increased risk of mortality (HR = 2.48) (7). Seventy-nine subjects (12.7%) were found to have liver disease in our cohort. Biopsy report was available in 40 subjects (Figure 2A). Amongst this group, granulomas (32.5%, *n* = 13) and nodular regenerative hyperplasia (NRH, 30%, *n* = 12) were the most common pathological features. Two patients were noted to have both granuloma and features of NRH. Nine subjects (22.5%) were noted to have general inflammation of the liver, with predominantly lymphoid infiltrates specified in 5 subjects. Primary biliary cholangitis was noted in 10% (*n* = 4), while primary sclerosing cholangitis was noted in 1 subject. Cirrhotic features predominated in 3 subjects at the time of biopsy.

Liver disease-associated sequelae were observed in 32.9% (*n* = 26) of the patients with known chronic liver disease (Figure 2B), and these may be severe with disease progression. These sequelae were equally likely to develop in either NRH or granulomatous liver disease in our cohort (two tailed *P* = 1.0, Fisher's exact test). Evidence of portal hypertension was seen in 32.9% (*n* = 26) of these subjects. Esophageal varices were found on endoscopy in 17.7% (*n* = 14), with variceal bleed recorded in 3 patients. Ascites was observed in 15.2% (*n* = 12), with spontaneous bacterial peritonitis noted in 3 subjects. Hepatopulmonary syndrome was recorded in 6.3% (*n* = 5), and all of the patients required chronic oxygen supplementation. Overt hepatic encephalopathy was noted in 2 subjects.

Five patients underwent liver transplant due to end stage liver failure. Clinical information, including primary liver disease, comorbidity, and outcome, was summarized in Table 6. Two patients (patient 1 and 2) suffered from acute rejection and died in the setting of organ failure and severe infections within 1 year. One patient (patient 3) died 2 years after transplant from complications associated with infections. Two patients are alive 3 years (patient 4) and 6 years (patient 5) after transplant. Patient 5 had recurrence of granulomatous disease in the transplanted liver, and was re-listed for a second liver transplant for stage 4 cirrhosis at the time of report.

**Table 6:** Liver transplant outcomes.

	**Patient 1**	**Patient 2**	**Patient 3**	**Patient 4**	**Patient 5**
Age, Sex	54, F	46, M	48, M	46, M	40, F
Liver pathology	Primary sclerosing cholangitis, cholangiocarcinoma	Hepatitis (non-A, non-B)^*^	Primary biliary cholangitis	Nodular regenerative hyperplasia	Granulomatous liver disease
CVID-associated conditions	ITP, AIHA	Chronic lung disease	Lung granulomas, splenomegaly	Bronchiectasis	AIHA, s/p splenectomy
Transplant procedure	Liver	Liver	Liver	Liver	Liver
Outcome	Died of acute rejection within 1 year	Died of acute rejection within 1 year	Died of infections after 2 years	Alive 3 years post-transplant	Alive 6 years post-transplant, recurrence of granulomas in transplanted liver

* *Before availability of hepatitis C virus PCR. AIHA, autoimmune hemolytic anemia; ITP, Immune thrombocytopenic purpura*.

### Lymphoma and Other Neoplastic Disease

Lymphoma in CVID has been shown to be associated with reduced survival (HR = 2.44) (7). Forty-two patients (6.7%) had a lymphoid malignancy in this cohort. Lymphoma was significantly more common in females (*n* = 30, 8.7% of female subjects) than males (*n* = 12, 4.3% of male subject; two-tailed *P* = 0.036, Fisher's exact test), as noted previously. Detailed pathology reports were available in 39 subjects (Table 7). All lymphomas recorded were B cell in type, with the exception that one patient had ALK negative anaplastic large cell lymphoma (Table 7). Amongst this group, the vast majority of lymphomas were non-Hodgkin's lymphoma (89.7%, *n* = 35). Hodgkin's disease was noted in 3 subjects (7.7%), and all 3 patients subsequently developed secondary lymphoma of unknown B cell type. Various types of B cell lymphoma were noted in the cohort, including diffuse large B cell lymphoma (*n* = 10), T cell rich B cell lymphoma (*n* = 3), plasmacytoid lymphoma (*n* = 1), marginal zone lymphoma (*n* = 5), and extranodal marginal zone lymphoma of MALT (*n* = 1). A few biopsies were categorized under the Working Formulation classification at the time of original pathology report, and they included diffuse mixed small and large cell lymphoma (*n* = 2), diffuse small cleaved cell lymphoma (*n* = 1), diffuse poorly differentiated lymphoma (*n* = 1), and follicular mixed cell lymphoma (*n* = 1). Ten patients had non-Hodgkin's lymphoma of B-cell type that was not further classified. Solid organ malignancies were seen in 40 subjects (6.4% of overall cohort, Table 8). Two patients developed 2 distinct primary malignancies (one subject with colon and prostate cancer, and one subject with prostate and skin cancer). One patient underwent allogenic hematopoietic stem cell transplantation (HSCT) for B cell lymphoma (not otherwise specified), but outcome data were unavailable.

**Table 7:** Lymphoma types.

	**No**.
**Detailed pathology available**	39
**Non-Hodgkin's lymphoma**	35
B-cell type, not otherwise specified^*^	10
Diffuse large B cell lymphoma	10
T cell rich B cell lymphoma^*^	3
Plasmacytoid lymphoma	1
Marginal zone lymphoma	5
Extranodal marginal zone lymphoma of MALT	1
Diffuse mixed small and large cell lymphoma	2
Diffuse small cleaved cell lymphoma	1
Diffuse poorly differentiated lymphoma	1
Follicular mixed cell lymphoma	1
Hodgkin's disease^**^	3
**Anaplastic large cell lymphoma (ALK -)**	1

* *One patient with +EBV in each category*.

** *All 3 patients subsequently developed secondary lymphoma of unknown B cell type*.

**Table 8:** Other cancers.

	**No**.
**Other cancers**	40
Breast cancer	9
Colon cancer	3
Lung cancer	3
Ovarian cancer	3
Gastric cancer	3
Melanoma	3
Cholangiocarcinoma	2
Oral cancer	2
Skin cancer	2
Thyroid cancer	2
Colon, prostate cancer	1
Esophageal cancer	1
Hepatic carcinoid tumor	1
Meningioma	1
Pituitary adenoma	1
Prostate, skin cancer	1
Testicular cancer	1
Vaginal cancer	1

### Granulomatous Disease and Lymphoid Proliferation

The morbidity and mortality impact of granulomatous disease in CVID may be dependent on its location. While the presence of granuloma in general was not previously associated with shorter survival, granulomas found in lungs and liver may lead to tissue destruction and organ-specific sequelae, as observed here and previously (7). These granulomatous changes may be mistaken as “sarcoidosis,” leading to delayed recognition of CVID. In prior reports, the association of granuloma, autoimmunity, and splenomegaly had been noted (8, 20). Granulomatous disease diagnosed by tissue biopsy was seen in 58 subjects (9.3% of overall cohort), and they typically consisted of well-formed non-caseating granulomas. This prevalence is likely an under-estimation as many patients did not undergo tissue biopsy. Granulomas may occur in a wide variety of organs (Table 9). The most common sites of granulomas identified by biopsies included lung (*n* = 25), liver (*n* = 13), skin (*n* = 10), and lymph nodes (*n* = 9). Rare but notable locations included brain (*n* = 2), bone marrow (*n* = 1), parotid gland (*n* = 3), and the mesentery (*n* = 1, presented as a large mesentery mass).

**Table 9:** Granulomatous disease by locations.

	**No**.
**Granulomas (total)**	58
Lung	25
Liver	13
Skin	10
Lymph node	9
Eye	3
Brain	2
Gastrointestinal tract	2
Oral	2
Parotid gland	2
Soft tissue	2
Spleen	2
Bone marrow	1
Kidney	1
Mesentery mass	1

Lymphoid hyperplasia and/or splenomegaly were common features, seen in 130 subjects (20.9% of overall cohort). Splenomegaly was recorded in 97 subjects, while lymphadenopathy was recorded in 56 subjects. Twenty-three subjects had concurrent splenomegaly and lymphadenopathy. Fifty-four patients (8.7% of overall cohort) underwent a splenectomy due to either uncontrolled cytopenias (ITP or AIHA) or hypersplenism. Amongst this group, 3 patients (5.6%) had documented sepsis due to an infection after splenectomy. Two additional patients had been hospitalized post-splenectomy for meeting clinical criteria for sepsis, though no positive microbiology was recorded. One of these 5 patients were not on immunoglobulin replacement at the time of sepsis. Rare additional complications had been noted in patients who had undergone splenectomy. As reported previously, 2 subjects developed fistulas to other organs or the exterior skin, and 2 subjects developed unexplained portal hypertension and secondary liver failure (7). Pulmonary arterial hypertension had previously been linked to post-splenectomy status in other diseases (29). Three out of 10 subjects with pulmonary arterial hypertension in our cohort had a history of splenectomy, though with concurrent chronic lung disease in all 3 patients, it was unclear whether splenectomy was a major contributing factor.

## Insights to the Pathogenesis of Non-Infectious Complications in CVID

### Monogenic Defects in Immune Regulation and B Cell Function

In the past 2 decades, genetic studies of the CVID phenotype have led to the successful identification of a number of monogenic defects (11–13). In our New York cohort, a monogenic etiology has been assigned to as many as ~30% of the subjects (12). Two broad categories of genetic defects have emerged from these studies. The first includes mutations in genes that are involved in various stages of B cell activation, survival, and maturation to the plasma cell stage. In the second category, defects in genes that control general immune regulation are also increasingly recognized in subjects with the CVID phenotype. In the later, additional features of autoimmunity and inflammation are commonly seen. Together, these monogenic defects have provided key insights to the molecular pathways that can lead to concurrent antibody deficiency and broader immune dysregulation in select patients, along with targeted therapies in some cases. The main genes linked to this syndrome and the associated clinical phenotype are discussed here.

Amongst immune regulatory genes, pathogenic variants of nuclear factor kappa B subunit 1 (NF-kB1) are the most common defects in the US and European CVID cohorts (12, 30). The NF-kB family of transcriptional factors includes 5 related proteins, c-Rel, p65 (RelA), RelB, p50 (NF-kB1), and p52 (NF-kB2), which forms various homo- and heterodimers to regulate the expression of a wide range of target genes (31). The NF-kB signaling pathway is involved in diverse processes, notably including B cell differentiation and function, as well as immune response to microbial and inflammatory stimuli. NF-kB1 defects identified in CVID cohorts exhibit autosomal-dominant inheritance with variable penetrance (32–35). These patients present with antibody deficiency and a B cell phenotype that is characteristic of CVID. Clinically, non-infectious manifestations are common, reflecting the broader impact of dysfunctional NF-kB signaling. These include autoimmunity, lymphoid hyperplasia, lung disease, enteropathy, liver disease, granulomas, and malignancy (30, 32–35). Autosomal dominant, heterozygous NF-kB2 defects have also been identified in CVID cohorts (36). These patients present with early onset hypogammaglobulinemia, infections, along with autoimmune features, and notably, adrenal insufficiency. Other non-sense gain-of-function NF-kB2 mutations have been identified in 2 families, characterized by hypogammaglobulinemia, lymphopenia, and T-cell defects (37).

Lipopolysaccharide (LPS)-responsive beige-like anchor protein (LRBA) and cytotoxic T lymphocyte antigen 4 (CTLA4) are closely associated proteins primarily known for the regulation of T cell response, but mutations in both genes have been identified in CVID cohorts (12). LRBA is localized in the vesicles and endoplastic reticulum, with domains homologous to vesicle trafficking proteins. Its function is closely linked to CTLA4 as it prevents lysosomal degradation of CTLA4 (38). As such, LRBA deficient patients have low CTLA4. CTLA4 is an inhibitory checkpoint protein on activated T cells and regulatory T (Treg) cells (39). It exerts immune-modulatory effects by competing with the T cell co-stimulatory molecule CD28 for the ligands CD80 and CD86 on antigen presenting cells (APC), or by removing these ligands by trans-endocytosis, resulting in reduced APC-mediated T cell activation. CTLA4 is essential for the function of Treg cells, which are responsible for maintaining self-tolerance and immune homeostasis. Homozygous recessive LRBA mutations have mostly been identified in childhood onset disease, but compound heterozygous mutations have been identified in adult CVID cohorts (40–42). In LRBA deficient patients, there are concurrent B cell defects (disturbed development, defective activation, poor plasmablast formation) manifesting as hypogammaglobulinemia, and T cell dysregulation (decreased Treg cell markers, expansion of T follicular helper cells, contraction of T follicular regulatory cells) that may contribute to inflammatory complications (38, 42). Clinically, these patients are affected by recurrent infections, autoimmunity, lymphoid hyperplasia, as well as severe inflammatory bowel disease in some (40–42). Heterozygous CTLA4 mutations, on the other hand, can lead to haploinsufficiency, impaired protein dimerization, or impaired ligand binding, causing an autosomal dominant syndrome with variable penetrance (43–46). Clinically, this highly variable syndrome is marked by antibody deficiency (84%), lymphoproliferation (73%), autoimmune cytopenia (62%), as well as lung (68%) and gastrointestinal (59%) diseases, amongst additional non-infectious complications, in a large cohort study (*n* = 133) (46). The elucidation of this molecular pathway and the availability of CTLA4 fusion proteins (abatacept, belatacept) have allowed for the potential for targeted treatment approach. In select cases, the loss of CTLA4 in LRBA deficiency has been successfully treated with abatacept (38). Similarly, some subjects with CTLA4 haploinsufficiency have had positive clinical response to abatacept or belatacept (11/14 subjects in one series) (46).

Phosphoinositide 3-kinase (PI3K) defect is another example of immune regulatory proteins that can lead to prominent antibody deficiency in the setting of broader dysregulation. PI3K contains multiple subunits, including the p110δ (PIK3CD) catalytic subunit and p85α (PIK3R1) regulatory subunit. PI3K is expressed predominantly in hematopoietic cells and is involved in signaling downstream of T and B cell receptors, toll-like receptors (TLRs), co-stimulatory receptors and cytokine receptors (47). It is therefore closely linked to the proliferation, survival, and activation of these cells. Dominant mutations in PI3KCD is now known as activated PI3Kδ syndrome (APDS), and some subjects may present with a CVID-like or hyper-IgM phenotype, with recurrent sinopulmonary infections, reduced IgG in 43%, reduced IgA in 50%, and increased IgM in 79% of subjects (48–51). This syndrome has incomplete penetrance and variable expressivity. Clinically, it is also characterized by a high prevalence of non-infectious manifestations, including lymphoproliferation (75%), autoinflammatory disease (34%), and lymphoma (13%) (51). However, unlike most CVID subjects, herpesvirus infection is relatively common (49%) in these patients, and their immunophenotype is notable for reduced naïve T cells, increased highly differentiated effector/effector memory T cells, and expanded transitional B cells (51). Dominant heterozygous mutations in the p85α (PIK3R1) regulatory subunit of PI3K have also been reported in 4 subjects from 3 families (52). These lead to a similar syndrome of hyperactive PI3K signaling, with reduced IgG and IgA, variable IgM, and recurrent sinopulmonary infections, along with lymphoproliferation, autoimmunity, and terminally differentiated effector T cells.

Other notable monogenic defects that can result in concurrent antibody deficiency and non-infectious complications include: signal transducer and activator of transcription 3 (STAT3) gain-of-function (GOF) germline mutations, inducible T-cell costimulatory (ICOS) deficiency, IKAROS deficiency, and an interferon regulatory factor-2 binding protein 2 (IRF2BP2) mutation. STAT3 is a transcription factor, regulating multiple processes that includes cellular proliferation, differentiation, as well as autoimmunity. Patients with STAT3 GOF mutations have prominent early-onset multiorgan autoimmune and lymphoproliferative features, though more than half of the subjects (18 of 28) also have hypogammaglobulinemia in a recent systematic review (53). Common non-infectious manifestations include hematologic autoimmunities, enteropathy, interstitial lung diseases, and endocrinopathies (53–55). ICOS is a surface receptor of T cells. It is in the same family of proteins as CD28 and CTLA-4, and it is required for both T cell-APC interaction and T cell-B cell interaction in the germinal center. Homozygous and compound heterozygous mutations in ICOS are overall rare, but can lead to non-infectious complications that include enteropathy, autoimmunity, and hepatomegaly (56, 57). IKAROS is a hematopoietic zinc-finger transcription factor that is considered as master regulators of lymphocyte differentiation. In addition to hypogammaglobulinemia that can be profound, 2 subjects in the original cohort had B-cell acute lymphoblastic leukemia and 1 subject had thrombocytopenic purpura (58). IRF2BP2 is a transcriptional co-repressor that can affect cytokine production and it may also be involved in the development or survival of memory B cells. In the original report, variable degree of Ig deficiency, enteropathy, psoriasis, and type 1 diabetes were observed in a family with an IRF2BP2 mutation (59). In select cases, hypomorphic mutations in recombinase activating genes (RAG1 and RAG2) may also lead to prominent autoimmunity/hyperinflammation and antibody defects; however, these patients often have atypical features, such as opportunistic infections or severe systemic viral infections, suggesting a diagnosis other than CVID (60–62).

Deficiencies in B-cell costimulatory molecules are relatively rare, but can lead to a CVID phenotype. These defects are not known for broad inflammatory complications, but select non-infectious complications have been noted in a few cases. CD19 is a transmembrane protein expressed throughout B cell development until the plasma cells stage. It forms a complex with CD21 and CD81 on the surface of mature B cells and together, they are involved in signal transduction through the B cell receptor. Autosomal recessive mutations in these genes, and also in CD20, lead to defective activation of B cells and hypogammaglobulinemia. Amongst these disorders, nephropathy and thrombocytopenia associated with anti-platelet antibodies have been reported in a child with a homozygous CD81 gene defect (63), while chronic diarrhea and splenomegaly was reported in an adult with CD21 deficiency (64). Other B cell surface receptors defects, such as mutations in the B-cell activating factor of the tumor necrosis family (BAFF) receptor (BAFF-R) (65, 66), which is involved maturation of splenic B cells, and autosomal recessive mutations in CD27 (67), a receptor associated with memory B cells, may also lead to a CVID phenotype. However, autoimmunity and inflammatory complications have not been reported in these patients.

Lastly, in some cases, mutations in B-cell-specific genes have been heavily associated with autoimmunity and inflammation in CVID, though the molecular pathways leading to such a phenotype are not as obvious. Transmembrane activator calcium-modulating cyclophilin ligand interactor (TACI) is a product of the gene TNFRSF13B, and it is a receptor found on B cells. It binds to B-cell activating factor (BAFF) as well as a proliferation-inducing ligand (APRIL), and supports class-switch recombination, plasma cell differentiation, and antibody secretion during later stages of B-cell development. TACI mutations, usually in the heterozygous state, are enriched in the CVID population (8–10%) (68, 69), and they are significantly associated with autoimmunity and lymphoid hyperplasia, implying an additional role of TACI in the establishment of tolerance (70–72). However, heterozygous TACI mutations can also be seen in the healthy population, albeit at a much lower frequency, and phenotypically “normal” relatives of CVID subjects (73). Thus, potential functional impact of heterozygous TACI variants remains an area of active investigation.

The complexity of genetic defects in those with the CVID phenotype continues to be revealed. There are currently no definitive answers for when genetic studies should be recommended. While many investigations have focused on subjects with non-infectious complications, monogenic defects have also been identified in as many as ~25% of those without in two large Western cohorts (*n* = 395 combined; in press). Disease severity may not necessarily correlate with the presence of a monogenic defect. In our cohort, no pathologic gene variants have been identified in patients with severe organ damage requiring transplantation. While there are no clear criteria, subjects with difficult-to-control autoimmunity, lymphocytic infiltrations in lungs/other organs, granulomatous disease, or lymphoma may benefit most from genetic investigations because such information may aid treatment selection. The clinical implication of monogenic defects for HSCT in CVID is currently not known; overall, HSCT rarely has been performed in this population, with mixed outcomes to-date (74).

### Immunologic Abnormalities Associated With Non-infectious Complications

In addition to the identification of monogenic defects in a subset of patients, broad adaptive and innate immune dysregulation are increasingly recognized in CVID, especially in those with non-infectious complications. In the B cell compartment, a number of phenotypic changes have been observed, and classifying patients based on this framework has found clinical relevance (21, 75). Amongst CVID subjects, the prominent B cell abnormality is a reduction of isotype-switched memory B cells, possibly reflecting defective germinal center development in this syndrome. Using a cutoff of ≤0.55% of peripheral B cells, Sanchez-Ramon et al. have shown that a severe lack of isotype-switched memory B cells in CVID is an independent risk factor for granulomas, autoimmune disease and splenomegaly (75). In some CVID patients, there is also an expansion of CD21^low^ B cells in the peripheral blood. These CD21^low^ B cells have previously been reported in other autoimmune states, carrying unique characteristics that include an unmutated B-cell receptor as well as potentially being polyreactive (76). In CVID, the expansion of CD21^low^ B cells (>10%) has been linked to splenomegaly (21). In a separate study, this phenotype is associated with autoimmune cytopenia as well (77). In another group of CVID subjects, an expansion of transition B cells has been identified. Notably, more than 50% of patients with this phenotype (defined as transitional B cells > 9%) has lymphadenopathy in one report (21). Lastly, in rarer cases, a near absence of B cells (<1% of total B cells) has been reported, suggesting severe defects of early B-cell differentiation in this group (21). An association between gender and distinct B cell profiles has also been described in CVID. In one study, female subjects are found to have higher levels of serum IgM and a greater number of isotype-switched memory B cells than male subjects (75), but whether this B cell phenotype differential is linked to the higher incidence of B-cell lymphoma in female CVID patients remains unknown.

In the T cell compartment, CVID is often characterized by broad and substantial T cell impairments, and in some cases, these defects are associated with overt clinical complications. As seen in our previous report (7), reduced T cell counts is observed in up to 29% of the cohort. In a minority (3.9%) of patients, severe CD4 T cell lymphopenia (<200 cu/mm) has been reported (7, 78). For these patients, an alternative diagnosis of late-onset combined immunodeficiency has been proposed as they can be prone to opportunistic infections (78). Functionally, impaired *in vitro* lymphocyte proliferation to both specific (antigen) and non-specific (mitogen) activators are observed in up to 50% of the CVID cohort (7). Impaired response to chemokines and abnormal lymphocyte trafficking have also been reported (79). Additional studies have shown that T cells in CVID often expresses activation markers and a marker associated with proliferation (Ki-67) (80); at the same time, they have a tendency to undergo apoptosis (81). However, the intrinsic and/or extrinsic drives behind these T cell impairments remain unclear. A reduction of regulatory T (Treg) cells, which are normally responsible for modulating immune response and maintain self-tolerance, has also been identified (77, 82). This phenotype is most profound in CVID subjects with reduced isotype-switched memory B cells and expanded CD21^low^ B cells. Clinically, decreased Treg cells are associated with lymphoproliferation and autoimmune cytopenia, along with elevated inflammatory markers (77, 82). Another pervasive T cell abnormality in CVID is a clonal and constricted T cell repertoire, but they do not appear to be associated with specific clinical complications based on the current literature (83).

A wide range of cytokine defects have also been described in CVID; some of these have been more directly linked to specific cellular defects and/or clinical compilations, and will be highlighted here. First, a lack of IL-2 production has long been observed in CVID, and this was thought to contribute to poor T cell proliferation and function in this disorder. In early clinical trials of recombinant IL-2 treatment, some clinical and immunologic benefits of this approach were indeed observed (84–86). In contrast, IL-7, which may contribute to proliferation of autoreactive T cell clones, appears to be elevated in a subgroup of CVID subjects (87). In this group, elevated serum IL-7 correlates with increased CD8+T cells and a higher incidence of autoimmunity, but is not associated with T cell lymphopenia (87). More recently, elevated serum B cell-activating factor (BAFF) have also been reported in CVID and it may drive pulmonary B cell hyperplasia in patients with progressive interstitial lung disease (88). Other cytokine alterations reported in CVID include increased IL-6 (89–91), increased TNF-α (92), and variable IL-4 (90, 91).

Using whole blood mRNA transcriptional profiling, Park et al. have reported a marked up-regulation of interferon responsive genes in CVID subjects with inflammatory complications when compared to those without such complications and from control subjects (93). At the same time, modular analysis of the RNA transcript showed a greater reduction of both B and T cell networks in those with inflammatory manifestations (93). Together, these observations point to an impaired adaptive immunity that is coupled with chronic activation of innate interferon pathways in CVID subjects with inflammatory complications. In the follow-up study, IFN-γ, IL-17A, and IL-22+ cells with markers of innate lymphoid cells type 3 (ILC3; lineage negative, CD127+, CD161+, T-box transcription factor+, and retinoid acid-related orphan receptor γ+) were found to be expanded in the peripheral blood of CVID subjects with inflammatory conditions (mean 3.7% of peripheral blood mononuclear cells) (94). These cells had inflammatory potentials and they were also identified in the gastrointestinal and lung tissues of CVID patients with non-infectious organ disease, suggesting a role in mucosal inflammation (94). An additional study has similarly identified an expansion of ILC3, most pronounced in CVID subjects with autoimmune and/or lymphoproliferative complications, but also a relative loss of ILC2 (95).

While broad immunologic abnormalities and inflammatory complications in CVID are likely intrinsic to the underlying genetic and immune defects, a potential influence of environmental stimuli, namely commensal bacteria and their products, has been the subject of ongoing investigation in recent years. Perreau et al. detected high levels of endotoxins in plasma of CVID subjects prior to starting Ig replacement therapy (96). This observation was associated with reduced proliferation capacity of bacteria-specific CD4 T cells and higher expression of programmed death 1 (PD-1) on CD4+ T cells, potentially suggesting a relatively “exhausted” phenotype. In this initial report, Ig replacement eliminated plasma endotoxin and reversed the CD4+T cells defects ascribed to the translocation of bacterial endotoxin (96). Thus, an alternative hypothesis for some CVID subjects, at least prior to Ig treatment, might be that chronic translocation of bacterial products could contribute to some levels of T cell impairment. Of note, the phenomenon of endotoxinemia in CVID has not been consistently detected in other reports, possibly due to the effects of Ig replacements, and it has not yet been directly linked to cellular activation (97, 98). At the same time, clinical data do not show that Ig therapy significantly alters the clinical course of most non-infectious complications (7). However, as we have come to better appreciate the bidirectional influence of host immunity and commensals on one another, and with increasing examples of their possible impacts in other autoimmune states (99, 100), the potential disturbance of host-commensal homeostasis and the associated immune consequences in CVID may warrant further evaluation.

## Conclusion

While CVID is classified among the B-cell defects, additional cellular defects and immune dysregulation have been recognized in this syndrome over time. This is reflected clinically in the broad spectrum of non-infectious manifestations seen in a significant proportion of patients, which can lead to further sequelae with disease progression and increased mortality compared to those without such complications. The introduction of immunoglobulin replacement therapy has reduced the incidence of severe respiratory tract infections and associated mortality seen in the early years. However, with a lack of effective treatment in many cases, chronic non-infectious inflammatory and autoimmune conditions have emerged as challenging clinical problems in CVID. Recent genetic studies of this phenotype have led to the identification of monogenic defects in both B-cell centric genes and broader immune regulatory genes, providing insights to pathogenesis and potentially more targeted treatments in select patients. Moving forward, further genetic and immunologic understanding of this complex and heterogeneous syndrome is needed for the development of new therapeutic approaches.

## Data Availability Statement

All datasets generated for this study are included in the article/supplementary material.

## Ethics Statement

The studies involving human participants were reviewed and approved by the Icahn School of Medicine at Mount Sinai Institutional Review Board. Written informed consent to participate in this study was provided by the participants or participants' legal guardian/next of kin.

## Author Contributions

HH and CC-R conceived the study, collected data, and drafted manuscript. CC-R provided critical revisions of the manuscript and final approval of the version to be published.

### Conflict of Interest

The authors declare that the research was conducted in the absence of any commercial or financial relationships that could be construed as a potential conflict of interest.
